# *Clostridium paraputrificum* Bacteremia and Ectopic Ileal Varices in Underlying Chronic Portal and Superior Mesenteric Vein Thrombosis: Report of a Rare Case

**DOI:** 10.1155/2024/9443664

**Published:** 2024-07-18

**Authors:** Grigorios Smanis, Eirini Avgoustou, Ioannis Papadopoulos, Antonios Papadopoulos, Athanasios Athanasakos, Elias Athanasiadis, Foula Vassilara

**Affiliations:** ^1^ 5^th^ Department of Internal Medicine Hygeia Hospital, Athens, Greece; ^2^ 4^th^ Department of Internal Medicine Medical School University Hospital “ATTIKON” National and Kapodistrian University of Athens, Athens, Greece; ^3^ Radiology Department Hygeia Hospital, Athens, Greece; ^4^ Oncologic Department Mitera Hospital, Athens, Greece

## Abstract

Ischemic bowel disease is considered a high-risk factor for infection from anaerobic bacteria, as the ischemic bowel is the perfect ground for their development. Herein, we present the case of an advance stage colon cancer patient with a rare cause of gastrointestinal bleeding and bacteremia due to *Clostridium paraputrificum*, a rare anaerobic Gram-positive bacterium. The patient had presented with several episodes of hematochezia in the context of chronic superior mesenteric-portal vein tumor thrombosis and rupture of ectopic varices, and the bacteremia was an unexpected complication of the bowel ischemia due to a combination of arterial ischemia and venous congestion.

## 1. Introduction

*Clostridium* species are anaerobic Gram-positive bacteria that form spores. They are part of the natural flora of the gastrointestinal tract, residing inside the mucosal barriers [1]. Clostridial species can cause infections that develop quickly and lead to severe clinical syndromes such as colitis, colonic necrosis, myonecrosis, bacteremia, and even death. An estimated bacteremia rate of 1.8/10^5^ per person per year is accredited to *Clostridium* spp. [2]. The most common *Clostridium* spp. causing bacteremia is *C. perfringens* (42%) followed by *C. septicum* (14%) and *C. ramosum* (9%) [1]. *Clostridium paraputrificum* is a rarely isolated clostridial species, and its clinical significance is not well studied.

Clostridia are almost always time-costly and difficult to identify timely in regular hospital practices. With the use of recent molecular methods such as in situ hybridization, fluorescent methods, and PCR techniques, isolation and identification of anaerobic bacteria have improved over the recent years [3, 4]. Recently, matrix-assisted laser desorption and ionisation time-of-flight mass spectrometry (MALDI-TOF MS) has been introduced as a standard method in diagnostic laboratories for quick and on-point identification of clostridia [5, 6].

Mesenteric vein thrombosis (MVT) is an infrequent condition and may be an acute, subacute, or chronic process. Chronic MVT may be an incidental finding or present with nonspecific abdominal symptoms delaying diagnosis [7]. Bleeding as a manifestation of chronic superior mesenteric vein thrombosis (SMVT) is a rare phenomenon and it should be considered in the differential diagnosis, when more common causes are excluded [8].

We report a rare case of bacteremia due to *C. paraputrificum* in a patient with advanced colon cancer and underlying chronic neoplastic MVT.

## 2. Case Presentation

An 88-year-old woman presented to the emergency department (ED) with severe gastrointestinal bleeding, in December 2022. She had a history of advanced ascending colon adenocarcinoma, first diagnosed in December 2021. She had undergone a robotic right colectomy. Postsurgically, 6 cycles of chemotherapy consisting of capecitabine 1.500 mg twice daily per os were completed. The cycle constituted of 2 weeks on treatment and one week off. Six months later, regarding her follow-up, a CT scan of her upper abdomen and pelvis with intravenous contrast material did not reveal any signs of relapse or superior mesenteric venous thrombosis.

**Figure 1:**
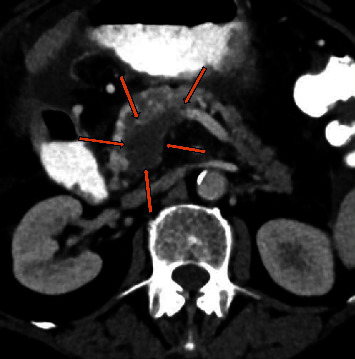
Computerized tomography (CT scan) portal vein phase, transverse section: a nonenhanced thrombus in the portal vein is depicted, indicating a chronic phase (orange arrows).

**Figure 2:**
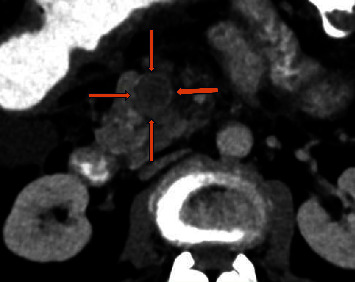
Computerized tomography (CT scan) portal vein phase, transverse section: an ill-defined hypodense lesion consistent with chronic thrombosis is depicted at the superior mesenteric vein (orange arrows).

**Figure 3:**
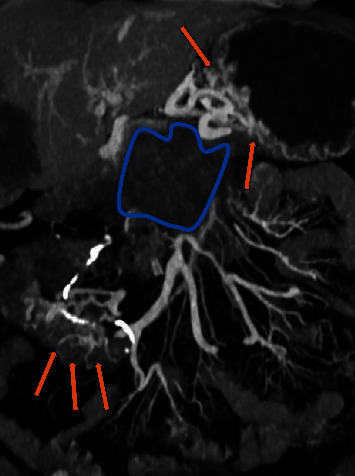
Computerized tomography (CT scan) portal vein phase, coronal view: ill-defined malignant tissue infiltrating the superior mesenteric vein and portal vein (blue outline). Dilated small veins compatible with collateral circulation (ectopic varices) in the terminal ileum (yellow arrows). Tortuous dilated veins are observed at the fundus of the stomach (orthotopic varices) (orange arrows).

At the presentation time at the ED, the patient had bouts of hematochezia, but she remained hemodynamically stable. A CT scan of the upper abdomen and pelvis was performed with intravenous contrast enhancement to investigate the cause of the bleeding. Surprisingly, an extensive thrombus to the superior mesenteric vein (SMV) and part of the portal vein was demonstrated, as well as metastatic liver disease. A neoplastic tissue at the anatomical proximity to excised ascending colon cancer, adjacent to the SMV, was visualized. Infiltration of the SMV by cancerous tissue was the most likely cause of thrombosis. The thrombosis of the SMV was complete, and collaterals to the small bowel and stomach had been already developed (Figures 1, 2, and 3). To further investigate the cause of bleeding, given the nonconclusive gastroscopy and colonoscopy, a capsule endoscopy was performed, and active bleeding was recognized in the terminal ileum. Scattered, probable angiodysplasias were visualized throughout the small bowel length.

**Figure 4:**
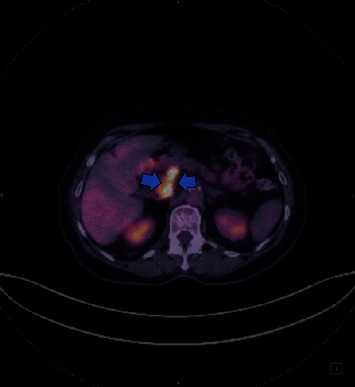
PET/CT transverse image: an area of linearly increased metabolic activity is observed in the upper abdomen, adjacent to the pancreas, which corresponds to a part of the portal vein and superior mesenteric vein consistent with thrombosis (neoplastic thrombosis) (blue arrows).

Due to active bleeding, anticoagulants were an absolute contraindication to treat thrombosis. Her treating surgeon and a vascular surgeon were consulted to discuss the possible options for this complicating and perplexing case. Given the chronic phase of thrombosis and the existence of venous collaterals, the vascular surgeon did not suggest any interventional thrombectomy. Her treating surgeon avoided a second surgery due to the high risk of complications. The patient was treated conservatively with red packed cell transfusions and fresh frozen plasma (FFP). When the patient recovered, she was referred to the oncology department. The metastatic liver disease was significantly responded to ensuing chemotherapy with 5-FU and oxaliplatin. However, the thrombus remained unchanged in the SMV, but partial recanalization of the portal vein was noticed over the course of her disease. At that time, a PET/CT was performed as an additional modality to secure the neoplastic nature of the thrombus (Figure 4).

In May 2023, she presented to the ED with pain and edema in the left calf. A Doppler-Triplex study revealed deep vein thrombosis of her left popliteal vein. Due to the anticoagulant therapy being contraindicated because of her past medical history, an inferior vena cava filter was placed. In September 2023, the patient was admitted to the hospital because of another episode of hematochezia. On admission, the patient was hemodynamically stable with a blood pressure of 110/65 mmHg, a pulse rate of 94 bpm, an oxygen saturation of 98% on ambient air, and a temperature of 36.5°C. On admission, the laboratory work-up showed a hemoglobin level of 9.0 g/dl that dropped as low as 5.4 g/dl over the next 3 days. Colonoscopy showed fragile and bleeding lesions at the site of the surgical anastomosis along with clots throughout the large bowel obscuring the mucosal visualization. Successful cauterization with argon plasma coagulation was performed at the anastomotic lesion. The patient was managed conservatively with blood transfusions, clotting factors, and daily administration of tranexamic acid 1 gm every 8 hours per day IV and was soon clinically stabilized. In this hospitalization, ascites was a prominent clinical finding which responded satisfactorily to diuretic therapy.

One week after admission, she presented a fever of 38.8°C with rigors and abdominal pain with peritoneal signs in the right lower quadrant. Laboratory work up revealed increased inflammatory markers (leukocytosis with neutrophilia and elevated CRP). Aerobic and anaerobic cultures were taken. Empiric antibiotic therapy with meropenem 2 gm every 8 hours daily and vancomycin 1 gm every 12 hours daily was initiated intravenously. The initial antibiotic regimen was upgraded because of the patient's previous hospitalizations. The anaerobic bacterium *C. paraputrificum* was isolated from the blood cultures very quickly thanks to the MALDI-TOF MS technique. In our laboratory, the identification of *C. paraputrificum* was performed using the MALDI-TOF MS (VITEK MS; bioMerieux, version 3.2) with a confidence value of 99.9. Sensitivity to metronidazole was reported using the classical Kirby–Bauer test.

As soon as this bacterium was isolated, the patient was covered with metronidazole 500 mg every 8 hours IV and ceftriaxone 2 gm once daily IV. Meropenem and vancomycin were discontinued. She immediately showed significant clinical improvement, remained afebrile, and the inflammatory markers returned soon to baseline values. At the current moment, the patient is in satisfactory condition.

## 3. Discussion

To the best of our knowledge, we present the first case of a patient with two rare manifestations: (1) the chronic neoplastic thrombosis of the SMV and portal vein leading to portal hypertension with ectopic ileal varices, as collaterals and (2) septicemia due to anaerobic microorganism *C. paraputrificum*.

Superior mesenteric vein thrombosis (SMVT) is classified as either primary or secondary, and most cases are associated with a malignant disease, an inflammatory process, a postoperative stage, and primary or secondary thrombophilia [7]. Pancreatic cancer is the most common neoplasm followed by colon cancer, as it happens to our patients. Treatment of SMVT varies considerably, and attention should be paid to characterize the stage properly. Acute SMVT and chronic SMVT are two separate clinical entities with different presenting symptoms and imaging studies. Acute SMVT is associated with a definite risk of bowel transmural ischemia and necrosis which may lead to perforation and peritonitis. Bleeding is a rare manifestation due to lack of collateral capillaries. CT scan depicts an enhanced rim around the thrombus without any collateral vessels. In contrast, in chronic SMVT, bleeding from varices at ectopic sites can happen because of the presence of collaterals. However, it constitutes only 5% of all variceal hemorrhages [9]. A triad of portal hypertension, hematochezia without hematemesis, and prior abdominal surgery characterizes the bleeding from small intestinal varices. All these findings corresponded perfectly to our patient [9, 10].

Most cases of acute SMVT can be treated with anticoagulation. Mechanical thrombectomy can provide rapid debulking but should be considered if the patient's condition deteriorates despite anticoagulation. Although a rare case of successful complete surgical resection of tumor thrombus in the SMV has been reported, it is not a common practice [10, 11]. The treating surgeon in our case did not adopt this technique. In our patient, the most difficult part of treatment was the massive hematochezia, which was an absolute contraindication in prescribing anticoagulants. Thankfully, in our patient, the adequate extensive vein collaterals protected her from fulminant catastrophic ischemia but brought her to the hospital several times for hematochezia due to rupture of ectopic ileal varices, which were successfully treated conservatively. 

*C. paraputrificum* is an anaerobic microbe that normally habituates the flora of the intestine and skin, and it is involved only in 1% of cases of *Clostridium* infection; therefore the precise morbidity and mortality rates due to infection of this species are largely unknown. A disruption of the mucus membrane that frequently occurs in ischemic bowel disease can facilitate the translocation of bacteria or their toxic constituents such as the lipopolysaccharides from the intestine directly into the bloodstream, leading to bacteremia. *C. paraputrificum* is rarely isolated from blood culture and only a few cases of bacteremia due to this microorganism have been reported in the past six decades, especially in patients with underlying conditions such as neutropenia, alcohol abuse, diabetes mellitus, sickle cell anemia, malignancy of pancreas and colon, and acquired immunodeficiency syndrome (AIDS) [12–15].

Our patient had the typical risk factors for septicemia of clostridial infection: (a) underlying relapsed right colon cancer (b) low-volume state because of massive hematochezia and shock (arterial hypoxia), and (c) chronic venous stasis and venous wall tension which occurs more easily at a lower pressure level in ectopic varices rather than in esophageal varices [9]. It is hypothesized that a rapidly growing tumor with anaerobic glycolysis provides an excellent environment enhanced by arterial hypoxia and venous stasis promoting clostridial germination. The right colon is considered the perfect environment for growth of clostridium spores. Given the reported association between clostridium infections and colon cancer, clinicians should be alert in investigating for colon adenocarcinoma, in every patient [16–18].

Clostridium infections are not uncommon, but most of the time, it is difficult to identify the exact species when the traditional culture methods are used. Accurate laboratory identification of *Clostridium* spp. is still a challenge, and other more specific methods, e.g., 16S RNA gene sequencing, are not used routinely because of their high cost and low availability [3, 4]. Over the last decade, MALDI-TOF MS has been demonstrated to be a rapid, accurate, and inexpensive alternative for the identification of anaerobic bacteria. A recent study demonstrated that the implementation of MALDI-TOF MS for the routine identification of anaerobes reduced the number of isolates that required DNA sequencing analysis for a conclusive species assignment to 3.1% (9/295). Besides, correct species identification was achieved in 85.8% of the cases and no misidentification at the genus level was detected. Given the enrichment of anaerobic reference spectra in the current database, it is expected more anaerobic species to be identified, which are unknown in clinical practice [19, 20]. This modality was pivotal for identifying *C. paraputrificum* in our case.

The most important point when treating a patient with *Clostridium* spp. infection is to start as early as possible the most effective antibiotic treatment depending on the isolate's susceptibility. A study of 138 cases of *Clostridium* species bacteremia revealed that 90% of these bacterial species were susceptible to penicillin and 99% of them were susceptible to metronidazole [1]. As for susceptibility to clindamycin, it was effective for 73% of the isolates. In this study, there were only two cases of *C. paraputrificum*; in both cases, the bacteria were susceptible to penicillin and metronidazole but resistant to clindamycin. This study highlighted the prognostic value of the timely start of empiric antibiotic treatment containing metronidazole to achieve the best clinical outcomes. Using only clindamycin as an empiric initial treatment is not indicated due to the higher resistance rates of *Clostridium* spp. [1].

## 4. Conclusion

Chronic portal vein and SMVT can create ectopic dilated vein varices due to the formation of a portosystemic shunt that are susceptible to rupture rather than the orthotopic varices. Ectopic variceal bleeding should be considered in all patients with portal hypertension who present with gastrointestinal hemorrhage in whom active bleeding or stigmata of recent bleeding are not evident on evaluation of the upper endoscopy. Ischemic bowel disease is a significant risk factor for infection from anaerobe bacteria. When patients present with hemorrhagic shock, the microcirculation of the small intestine is disrupted the most and the mucosal barrier is ruptured facilitating the entrance of pathogens to systemic circulation.

*C. paraputrificum* is a rare *Clostridium* species which has scarcely been reported to cause bacteremia when it penetrates the mucosal barrier of the necrotic bowel directly into the bloodstream. MALDI-TOF MS technique has shown great promise for fast, reliable, and cost-effective identification of clostridial infections. Newer pathogens might not be truly new, such as *C*. *paraputrificum,* but novel techniques such as MALDI-TOF MS may reveal preexisting but unknown pathogens. The exact pathogenicity of these novel microorganisms will be unveiled as more data are accumulated upon this topic.

Treatment with metronidazole should be considered in all patients with *Clostridium* bacteremia until antibiotic susceptibility is determined to minimize the risk of treatment failure. Underlying bacteremia from any pathogen that is part of the normal intestinal flora should alert the clinicians to investigate for occult colon cancer.

## Data Availability

All the necessary data and information used to support the findings of this study are included within the article.

## References

[1] Cohen-Poradosu R., Kasper D. L., Bennet J. E., Dolin R., Blaser M. J. (2020). Anaerobic infections: general concepts. *Mandell, Douglas and Bennett’s Principles and Practice of Infectious Diseases*.

[2] Leal J., Gregson D. B., Ross T., Church D. L., Laupland K. B. (2008). Epidemiology of *Clostridium* species bacteremia in Calgary, Canada, 2000-2006. *Journal of Infection*.

[3] Wildeboer-Veloo A. C. M., Harmsen H. J., Welling G. W., Degener J. E. (2007). Development of 16S rRNA-based probes for the identification of Gram-positive anaerobic cocci isolated from human clinical specimens. *Clinical Microbiology and Infection*.

[4] Song Y. (2005). PCR-based diagnostics for anaerobic infections. *Anaerobe*.

[5] Veloo A. C., Welling G. W., Degener J. E. (2011). The identification of anaerobic bacteria using MALDI-TOF MS. *Anaerobe*.

[6] Veloo A. C., Knoester M., Degener J. E., Kuijper E. J. (2011). Comparison of two matrix-assisted laser desorption ionisation-time of flight mass spectrometry methods for the identification of clinically relevant anaerobic bacteria. *Clinical Microbiology and Infection*.

[7] Kumar S., Sarr M. G., Kamath P. S. (2001). Mesenteric venous thrombosis. *New England Journal of Medicine*.

[8] Flynn K., Chung K., Brooke T., Keung J. (2022). Ectopic variceal bleeding from chronic superior mesenteric vein thrombosis after hemorrhagic pancreatitis. *Clinical Case Reports*.

[9] Tranah T. H., Sayagam J., Gregory S. (2023). Diagnosis and management of ectopic varices in portal hypertension. *The Lancet Gastroenterology & Hepatology*.

[10] Otani K., Ishihara S., Hata K., Murono K., Sasaki K., Yasuda K. (2018). Colorectal cancer with venous tumor thrombosis. *Asian Journal of Surgery*.

[11] Yanagida Y., Amano T., Akai R., Toyoshima A., Kobayashi J., Hashimoto T. (2020). Treatment of tumor thrombus in the superior mesenteric vein due to advanced colon cancer with complete surgical resection and chemotherapy: a case report. *Surg Case Rep*.

[12] Brook I., Gluck R. S. (1980). Clostridium paraputrificum sepsis in sickle cell anemia. *Southern Medical Journal*.

[13] Rodríguez R. T., Solís Marquínez M. N., Álvarez M. K. C. (2023). *Clostridium paraputrificum* bacteremia in a 64-year-old woman with colon carcinoma. *Anaerobe*.

[14] Hosin N., Abu-Ali B. M., Al Rashed A. S., Al-Warthan S. M., Diab A. E. (2023). *Clostridium paraputrificum* bacteremia in a patient with human immunodeficiency virus infection. A case report and literature review. *Infection and Drug Resistance*.

[15] Kwon Y. K., Cheema F. A., Maneckshana B. T., Rochon C., Sheiner P. A. (2018). Clostridium paraputrificum septicemia and liver abscess. *World Journal of Hepatology*.

[16] Mizza N. N., McCloud J. M., Cheetham M. J. (2009). Clostridium septicum sepsis and colorectal cancer- a reminder. *World Journal of Surgical Oncology*.

[17] Khan A. A., Davenport K. (2006). A reminder of the association between Clostridium septicum and colonic adenocarcinoma. *International Seminars in Surgical Oncology*.

[18] Laupland K. B., Edwards F., Furuya-Kanamori L., Paterson D. L., Harris P. N. A. (2023). Bloodstream infection and colorectal cancer risk in queensland Australia 2000-2019. *The American Journal of Medicine*.

[19] Alcala L., Marin M., Ruiz A. (2021). Identifying anaerobic bacteria using MALDI-TOF mass spectrometry: a four-year experience. *Frontiers in Cellular and Infection Microbiology*.

[20] Rodriguez-Sanchez B., Alcala L., Marin M., Ruiz A., Aloso E., Bouza E. (2016). Evaluation of MALDI-TOF MS (matrix- assisted laser desorption ionization time- of- flight mass spectrometry) for routine identification of anaerobic bacteria. *Anaerobe*.

